# High-Speed Electro-Optic Modulators Based on Thin-Film Lithium Niobate

**DOI:** 10.3390/nano14100867

**Published:** 2024-05-16

**Authors:** Songyan Hou, Hao Hu, Zhihong Liu, Weichuan Xing, Jincheng Zhang, Yue Hao

**Affiliations:** 1 Guangzhou Institute of Technology, Xidian University, Guangzhou 510555, China; zhliu@xidian.edu.cn (Z.L.); xingweichuan@xidian.edu.cn (W.X.); 2 State Key Laboratory of Wide Bandgap Semiconductor Devices and Integrated Technology, School of Microelectronics, Xidian University, Xi’an 710071, China; yhao@xidian.edu.cn; 3 National Key Laboratory of Microwave Photonics, Nanjing University of Aeronautics and Astronautics, Nanjing 211106, China; hao.hu@nuaa.edu.cn

**Keywords:** electro-optic modulator, lithium niobate, waveguide, integrated nanophotonics

## Abstract

Electro-optic modulators (EOMs) are pivotal in bridging electrical and optical domains, essential for diverse applications including optical communication, microwave signal processing, sensing, and quantum technologies. However, achieving the trifecta of high-density integration, cost-effectiveness, and superior performance remains challenging within established integrated photonics platforms. Enter thin-film lithium niobate (LN), a recent standout with its inherent electro-optic (EO) efficiency, proven industrial performance, durability, and rapid fabrication advancements. This platform inherits material advantages from traditional bulk LN devices while offering a reduced footprint, wider bandwidths, and lower power requirements. Despite its recent introduction, commercial thin-film LN wafers already rival or surpass established alternatives like silicon and indium phosphide, benefitting from decades of research. In this review, we delve into the foundational principles and technical innovations driving state-of-the-art LN modulator demonstrations, exploring various methodologies, their strengths, and challenges. Furthermore, we outline pathways for further enhancing LN modulators and anticipate exciting prospects for larger-scale LN EO circuits beyond singular components. By elucidating the current landscape and future directions, we highlight the transformative potential of thin-film LN technology in advancing electro-optic modulation and integrated photonics.

## 1. Introduction

High-speed electro-optic modulation is fundamental to numerous critical applications spanning optical communication [1], microwave photonics [2], computing [3], frequency metrology [4,5], and quantum photonics [6,7]. Various methods have been employed for electro-optic modulation, including carrier plasma dispersion [8], electro-absorption [9], and the Pockels effect [5]. The latter is particularly compelling due to its ability to offer ultrafast and pure refractive-index modulation across an exceptionally broad optical spectrum without introducing additional dissipation loss [5]. Prominent electro-optic modulators have been widely achieved in various platforms including silicon [8], silicon nitride [10], gallium arsenide [11], indium phosphide [12] and aluminum nitride [13]. Despite significant advancements photonic integration in these platforms, they have not yet demonstrated the capability to simultaneously realize ultralow propagation loss, rapid and low-loss optical modulation, and efficient all-optical nonlinearities. For example, although large-scale and low-cost photonic chips with silicon photonics have been commercially available due to the mature complementary metal–oxide–semiconductor (CMOS) technology, the lack of intrinsic electro-optic effect in silicon prevents achieving high bandwidth and low power consumption. Lithium niobate (LiNbO_3_, LN) stands out as one of the most prominent electro-optic Pockels materials, extensively utilized in telecommunications. Recently, thin-film monolithic LN has emerged as a promising platform, as this platform combines low-loss, high-quality photonic integration with the robust Pockels effect, enabling superior modulation performance [1]. With its potential to serve as an outstanding medium for photonic integrated circuits and future photonic interconnects, thin-film monolithic LN holds significant promise for advancing electro-optic modulation and integrated photonics [14].

This review endeavors to provide a comprehensive overview of integrated electro-optic modulators utilizing thin-film lithium niobate (LN), spanning from foundational principles to cutting-edge developments. Among LN’s remarkable properties, the electro-optic (EO) effect stands out as particularly enticing. This effect facilitates the direct integration of optical and RF fields, enabling a plethora of functionalities including optical modulation, sideband generation, and frequency shifting within the gigahertz range. The implementation of low-loss ridge waveguides coupled with closely spaced microwave electrodes, robust field confinement, and high-quality resonators in thin-film LN has significantly enhanced the EO interaction strength. Recent advancements have witnessed a surge in research on EO modulators in LN, leveraging both non-resonant and resonant optical structures to achieve broader microwave bandwidths and lower half-wave voltages. Exploiting this efficient EO interaction has also led to the development of EO frequency combs, holding promise for applications in spectroscopy and topological photonics. In the realm of quantum technologies, EO-based frequency conversion emerges as a compelling avenue to bridge microwave and optical photons, with potential implications for superconducting quantum systems and long-haul optical networks. Furthermore, the utilization of time modulation facilitates the exploration of synthetic dimensions in the frequency domain. This section comprehensively covers EO optical modulators, EO frequency comb sources, coupled-ring modulators (photonic molecules), cavity EO for quantum transduction, and EO-modulation-based synthetic photonics, elucidating their significance and potential contributions to the field.

## 2. Photonic Properties of Thin-Film Lithium Niobate

**Figure 1:**
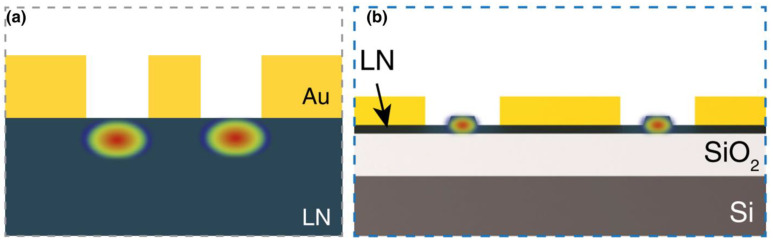
Optical mode distribution in LN waveguides. (**a**) Mode profile in bulk LN with Ti in-diffusion waveguide. (**b**) Mode profile in waveguide in thin-film LN. Adapted from [15].

LN boasts a wide transparency window spanning from 350 nm to 5 µm, encompassing the visible, near-infrared, and mid-infrared wavelength ranges. With a relatively large refractive index (~2.2 at 1550 nm), LN facilitates the formation of high-index-contrast waveguides on various substrates (see Figure 1), including amorphous and crystalline ones such as SiO_2_ or sapphire. Its high Curie temperature (~1210 °C) ensures the stability of its ferroelectric phase, rendering it compatible with a diverse array of fabrication processes and operating conditions. In contrast to Si and SiN_x_, LN processes large second-order nonlinear coefficients with d_33_ = 27 pm/V and remarkable Pockels coefficient (r_33_ = 31 pm/V), which makes LN-based modulators at the pivotal components in optical communication networks. The comparison of photonic properties between LN and other popular materials is listed in Table 1.

Traditional waveguides in bulk LN have historically relied upon two primary fabrication methods: titanium (Ti) in-diffusion and the proton exchange technique. However, the low optical index contrast between bulk LN waveguide and surroundings is a significant limitation for integration (Figure 1a), which leads to inadequate optical confinement with the large mode size and large bending radius in the millimeter range. Consequently, this weak optical confinement in bulk LN poses challenges in dense integration, microresonators and dispersion engineering.

**Figure 2:**
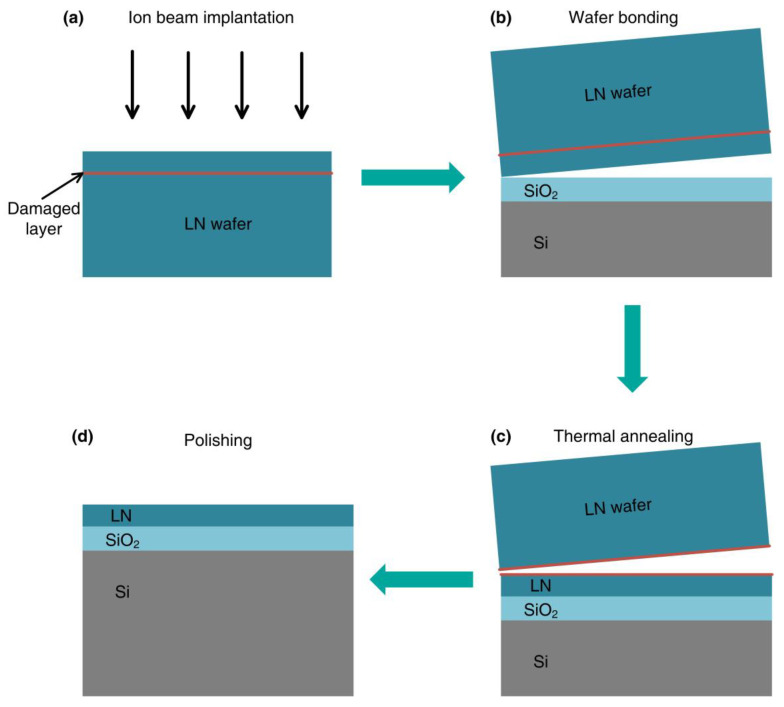
LNOI wafer fabrication using Smart-Cut technology.

Compared to bulk LN counterparts, thin-film LN platforms not only retain the excellent material properties of LN but also offer significantly improved light confinement, integrability, and compactness due to a substantial increase in refractive index contrast (Figure 1b). This merit is particularly noteworthy as it equips the LN platform with a comprehensive array of high-performance devices, including broadband frequency comb sources, ultra-high-Q microresonators, programmable filters, efficient frequency converters/shifters, and low-loss delay lines. Importantly, these devices hold the potential for integration with high-linearity electro-optic (EO) modulators on the same photonic chip, enabling the realization of complex microwave photonics (MWP) functions. Notably, a multitude of miniaturized and high-performance thin-film LN modulators have already been demonstrated, showcasing ultra-high bandwidths surpassing 110 GHz, which works with CMOS-compatible drive voltages, and lossless waveguides. Recently, high-quality thin-film LN-on-insulator (LNOI) wafers were produced using “Smart-Cut” technology, which is commonly employed for the fabrication of Silicon-On-Insulator (SOI) wafers. The fabrication of LNOI using Smart-Cut technology is illustrated in Figure 2. Starting with a high-quality LN bulk wafer, a damaged interplane is first defined through helium (He^+^) or hydrogen (H^+^) ion implantation. Simultaneously, the SiO_2_/Si substrate wafer is prepared with an adhesive layer. Then, the bulk LN with a damaged layer is bonded to the substrate wafer and followed by the thermal annealing process to split the LN substrate along the damaged layer, leaving thin-film LN bonded to SiO_2_/Si substrate. The subsequent annealing process is utilized to alleviate crystal defects induced by the ion collision, while the surface smoothness is improved by the chemical mechanical polishing (CMP).

## 3. Etching of LN Waveguides

**Figure 3:**
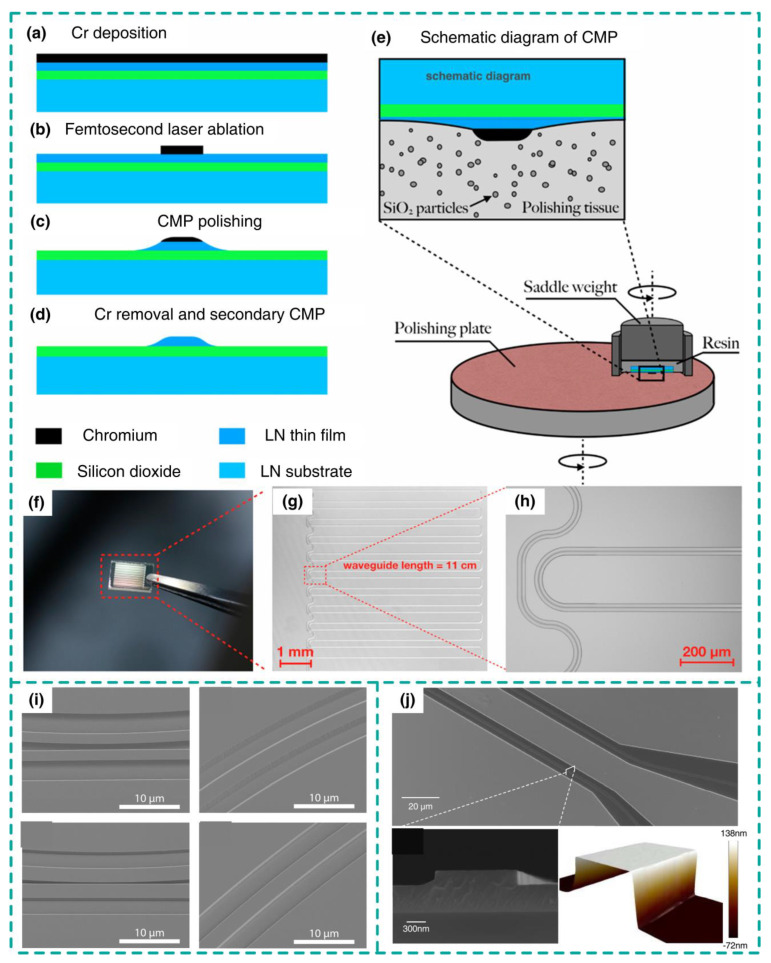
LN waveguides fabricated by CMP polishing and wet etching. (**a**–**d**) Schematic of LN waveguides through CMP. (**e**) Diagram of CMP. (**f**–**h**) Characterization of CMP fabricated LN waveguides. (**i**,**j**) Results of LN waveguides through wet etching. (**a**–**h**) Adapted from [16]. (**i**) Adapted from [17]. (**j**) adapted from [18].

The LN waveguides can be patterned through mechanical polishing, wet etching and dry etching. As shown in Figure 3, the chemical mechanical polishing (CMP) process basically involves four steps [16]: chromium deposition; mask patterning through laser ablation or lithography; CMP polishing; chromium removal with post polishing. LN waveguides with sub-nanometer roughness yield an optical loss of 0.027 dB/cm, enabling the realization of high-density photonic integration. However, LN waveguides fabricated through CMP are accomplished with shallow sidewalls, imposing a challenge on the bending radius. Wet etching with a solvent consisting of H_2_O_2_, NH_4_OH and H_2_O has also been employed to fabricate LN waveguides [17,18]. As shown in Figure 3i,j, high-quality LN waveguides with smooth sidewalls have been demonstrated using wet etching technology, which yields an intrinsic quality factor as high as 10^7^ and a large EO bandwidth of 110 GHz. The advantages of wet etching technology lie in their cost-effectiveness, high reproducibility and high efficiency, which also avoid EBL-induced sidewall roughness from photoresist. However, given the crystal anisotropic property of LN, the wet etching process must be controlled carefully to get rid of crystal defects, which will be further amplified during etching. The anisotropic etching rate of wet etching limits its capability to fabricate symmetrical and high aspect ratio LN waveguides.

In contrast to silicon and silicon nitride platforms, the absence of a suitable reactive ion etching recipe for LN opens up challenges for dry etching. The non-volatile lithium fluoride (LiF) redeposition is formed during the fluorine-based dry etching of LN, which stops further etching and results in sidewall roughness. To avoid LiF redeposition, Ar^+^ plasma-based physical dry etching is preferred for LNOI structure fabrication. Compared to CMP and wet etching, the etch depth can be well controlled with a symmetric cross-section. There are several challenges associated with the physical dry etching of LN. First, considering the low selectivity of etched materials and available photoresists, hydrogen silsesquioxane (HSQ) [6,19,20,21,22,23,24], Zeon electron-beam positive-tone resist (ZEP) [25], CSAR [26], SU8 [27], as well as hard marks including Cr and Si are commonly used. The second challenge is nonvertical sidewalls with an angle ranging from 40° to 80°, which results from LN redeposition during dry etching. This redeposition and other contaminants can be removed by the wet etching process. The domain optical loss in Ar^+^ etched LN waveguides is attributed to sidewall roughness. To improve the sidewall roughness, the dry etching parameters have to be optimized. LN waveguides with a low loss of 0.1 dB/cm have been demonstrated by several groups, which proves that thin-film LN is a reliable photonic platform. Alternatively, the sidewall roughness can also be reduced by the CMP after the dry etching of LN [28].

## 4. Non-Resonant Electro-Optic Modulators Based on Thin-Film LN

**Figure 4:**
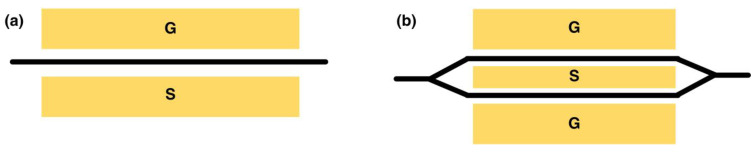
Common LN modulators. (**a**) Phase modulator, where the index of the waveguide is modulated by the applied voltages. (**b**) MZI intensity modulator, consisting of two phase modulators experiencing opposite phase changes. G: Ground electrode, S: Signal electrode.

EO modulators, converting electrical waves into light waves, play a pivotal role in current communication systems. Among various candidates, LN has been widely preferred for host material of modulators due to its substantial EO coefficient, chemical stability, and low RF and optical losses. While bulk LN-based modulators have been commercially available for decades, transitioning EO modulators into thin-film platforms brings up unique opportunities for advanced photonic integration [29,30,31]. The essential of the LN electro-optic modulator relies on the modification of the refractive index of LN crystal by an internal electric field. Figure 4 shows typical EO modulators in x- or y-cut LN. The simplest type is a phase modulator with only one Pockels cell (Figure 4a), where the phase delay of transmitted light is induced by the external voltages. The alignment between the polarization of input light and extraordinary axes of LN crystal is required for robust modulation efficiency. Combined with two-phase modulators as shown in Figure 4b, typical Mach–Zehnder interferometer (MZI) based modulators consist of two arms with opposite polarities and a coplanar waveguide (CPW) electrode, yielding efficient modulation of optical signals in accordance with microwaves.

**Figure 5:**
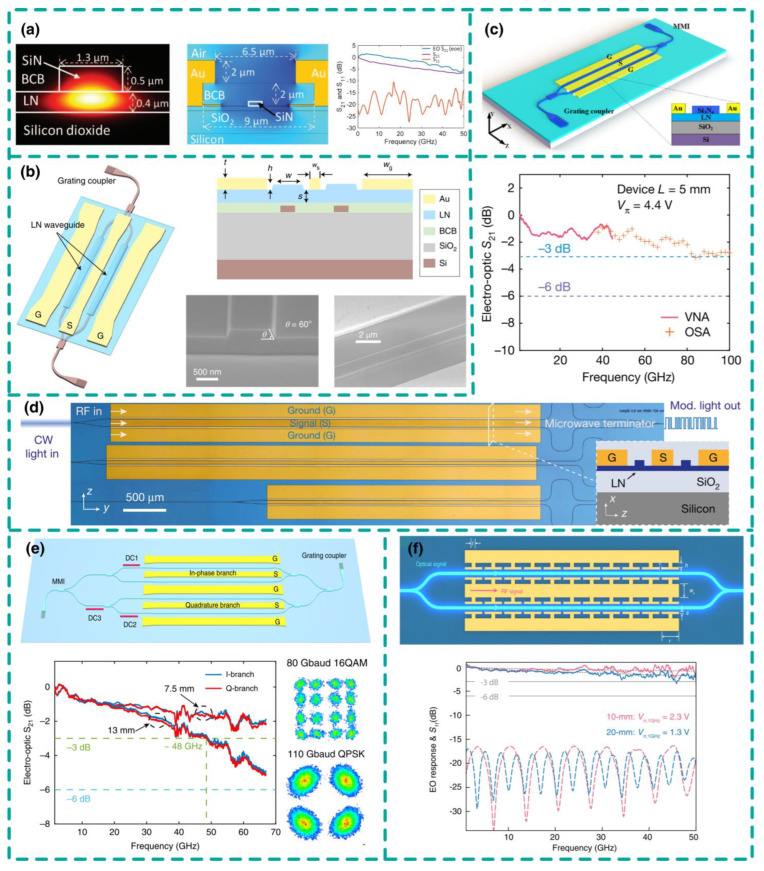
Representative types of nonresonant thin-film LN modulators. (**a**) Heterogeneously integrated Si_3_N_4_/LN modulators with Si_3_N_4_ as guiding waveguides on top of thin-film LN. (**b**) Heterogeneously integrated LN modulator on silicon on insulator (SOI) platform. (**c**) Low loss Si_3_N_4_/LN modulator. (**d**) Monolithic LNOI monolithic modulator based on Mach–Zehender interferometer (MZI). (**e**) Coherent in-phase and quadrature (IQ) modulator on LNOI platform with a working bandwidth over 70 GHz at 7.5 mm length. (**f**) LNOI modulator with structured electrodes, showing a bandwidth of 50 GHz with a V_π_ of 1.3 V in a 20 mm device. (**a**) Adapted from [33], (**b**) adapted from [35], (**c**) adapted from [37], (**d**) adapted from [1], (**e**) adapted from [45], (**f**) adapted from [46].

In order to reduce the optical loss of waveguides and bypass the challenges of LN direct dry etching, the research focus has gradually shifted towards hybrid/heterogeneous integrated devices based on the LNOI platform. In 2013, the research group led by S. Fathpour demonstrated an electro-optic modulator based on heterogeneously integrated silicon nitride ridge waveguides on the LNOI platform [32]. The modulator operated with a half-wave voltage of 6.8 V and a V_π_·L modulation efficiency of 4 V·cm, significantly exceeding typical commercial LN modulators. By optimizing the traveling-wave electrode of this heterogeneous integrated structure, the same research group achieved a half-wave voltage of 3.9 V and a 33 GHz operating bandwidth on the LNOI platform in 2016, with an optical transmission loss of 1.2 dB/cm [33]. This enhancement extended the significantly improved DC modulation efficiency to the radio frequency range (see Figure 5a). Furthermore, by employing a reverse ridge design in LN, the modulator achieved a 110 GHz operating bandwidth [34], albeit with a high transmission loss of up to 7 dB/cm. In 2020, Xinlun Cai’s team from Sun Yat-sen University proposed a hybrid silicon/LN waveguide structure on a silicon-based chip (see Figure 5b), capitalizing on the low-cost and high flexibility of silicon combined with LN’s excellent electro-optic properties [35]. They achieved modulation frequencies exceeding 70 GHz and a modulation efficiency of 2.2 V·cm using the high-performance modulation of the upper thin-film LN waveguide. Simultaneously, they utilized the lower-layer high-quality silicon waveguide for low-loss optical transmission, with an insertion loss of less than 2.5 dB. Efficient optical transmission between waveguides was achieved through vertical adiabatic couplers. Additionally, the device exhibited high linearity and integration, with a switch modulation rate of up to 100 Gbit/s and an amplitude modulation rate of 112 Gbit/s. In 2021, the research group led by Yi Luo and Bing Xiong from Tsinghua University designed a SiO_2_-LNOI hybrid waveguide with an electrode spacing of only 3 μm [36]. By adjusting the SiO_2_ thickness, they achieved the phase matching between microwave and optical waves, enabling a half-wave driving voltage of 3.4 V and demonstrating a modulation bandwidth of up to 67 GHz within a 5 mm modulation region. Also, in the same year, Yonghui Tian’s team from Lanzhou University prepared a silicon nitride waveguide on the LNOI platform, as shown in Figure 5c. This modulator fully utilized LN’s excellent electro-optic modulation performance while avoiding dry etching of LNOI, achieving a 3 dB modulation bandwidth of 30 GHz and a modulation efficiency of 2.24 V∙cm, with an extinction ratio of approximately 20 dB, and a switch key-controlled modulation speed of 80 Gb/s [37]. Building upon Si-LN heterogeneous integration, Professor Liu’s group from Zhejiang University further improved the performance of the modulator by employing a capacitive load-type traveling-wave electrode design based on substrate trenching, achieving a half-wave voltage of 1.7 V and a working bandwidth of over 67 GHz [38]. Meanwhile, heterogeneous integrated detectors and lasers based on the LNOI platform are also gradually developed [39], as summarized in Table 2. Heterogeneous integrated LNOI modulators have demonstrated high-performance electro-optic modulation. However, heterogeneous/hybrid integration faces a series of challenges such as material mismatch, high optical loss, and high packaging costs. Addressing these issues requires in-depth research in materials science, photonic chip design, manufacturing processes, and packaging technologies to achieve high-performance, stable, and cost-effective photonic chip heterogeneous integration.

**Table 2:** Performance of heterogeneous/hybrid LN modulators.

Material Platforms	V_π_·L (V∙cm)	3 dB Bandwidth (GHz)	Optical Propagation Loss (dB/cm)
LN on SOI [35]	2.55	70	0.98
Sulfide/LN [40]	3.8	1	1.2
Ta_2_O_5_/LN [32]	4	-	5
SiN on LN [41]	3	8	7
SiN on LN [33]	3.1	33	-
SiN on LN [42]	1.925	-	<2.25
LN on SiN [43]	6.67	30.55	1.6
LN on SOI [44]	6.7	>106	0.6

The scheme of electro-optic modulators based on monolithic integration on the LNOI wafer has become a hot research topic due to its scalability, and it has significant applications in fields such as telecommunication systems and optical networks, leading to numerous studies and applications in this area. With the breakthrough in low-loss LNOI waveguide etching technology [19,47,48], single-chip LNOI modulators have demonstrated higher modulation efficiency, while microwave design and device fabrication have become more convenient. In 2018, Harvard University demonstrated a single-drive high-performance LNOI electro-optic modulator by optimizing the traveling-wave electrode design and LN etching process (see Figure 5d). The half-wave voltage of the modulator was only 1.4 V, and the 3 dB operating bandwidth reached 45 GHz, with an internal optical transmission loss of approximately 0.5 dB [1]. In 2020, Xinlun Cai’s team from Sun Yat-sen University demonstrated a more complex IQ modulator for coherent communication on a single-chip LNOI platform (see Figure 5e). The performance level of each IQ branch was similar to that of a single modulator. The modulation rate of the device reached 320 Gbit/s, far exceeding that of commercial LN modulators [45]. To further improve the operating bandwidth of LNOI modulators, researchers have proposed more complex designs for the modulator’s electrode structure. To overcome the dominant Ohmic losses in transmission line electrodes, several design concepts are being explored, including thick electrodes and asymmetric electrodes. For instance, as shown in Figure 5f, researchers at HyperLight in the United States designed an electrode structure with periodic slotting slow waves on the LNOI platform [46]. Over a modulation length of centimeters, a wide range of speed and impedance matching was achieved, with a 3 dB modulation bandwidth reaching 110 GHz, breaking through the voltage-bandwidth limitation. When the frequency approaches or exceeds 100 GHz, other RF loss sources should also be considered, including linear dielectric absorption loss and substrate radiation loss. In LNOI modulators, the substrate radiation loss is significantly suppressed due to the reduced electrode gap, thereby efficiently reducing microwave losses. Table 3 summarizes the monolithic LNOI modulator performance. Bulk LN modulators face strong attenuation from substrate radiation above 70 GHz, while LNOI modulators have demonstrated modulation rates of up to 500 GHz. Compared to bulk LN modulators, LNOI modulators also have advantages in size. The size of LNOI modulators is limited by V_π_·L, i.e., the size requirement to achieve a specific driving voltage. Currently, the V_π_·L of LNOI modulators is 1.5–3 V·cm. Significantly improving V_π_·L without significantly increasing optical and microwave losses poses a significant challenge. For LNOI modulators, the V_π_ of a modulator with a length of 5 mm is approximately 4 V, but its bandwidth exceeds 100 GHz. In contrast, the V_π_ of a silicon modulator with the same size is approximately 6.3 V, with a bandwidth of 30 GHz and a phase shifter loss of over 5 dB. Therefore, to fully exploit the low-driving-voltage characteristics of LNOI modulators (such as V_π_ is approximately 1 V), the electrode length needs to reach 20 mm [42]. Due to the spatial constraints of V_π_·L improvement, to achieve a balance between bandwidth and voltage performance, the effective region length of MZI-type LNOI modulators may range from the millimeter to low centimeter.

## 5. Resonant Electro-Optic Modulators Based on Thin-Film LN

With the rapid advancement of micro-nano device processing technology, the size of detectors and laser chipsets in large-scale photonic links has reached the micron level, while the size of direct-waveguide modulators based on MZI is usually in the millimeter range (over 5 mm), greatly limiting the development of miniaturization of photonic integrated circuits. At the same time, the transmission of light in long-distance waveguides will lead to phase mismatch, requiring additional phases to compensate for the phase difference between different devices, further increasing the size of the devices. To further enhance the optoelectronic coupling, improve the efficiency of electro-optic modulators, and reduce device size, resonant modulators based on Bragg gratings, photonic crystals, and micro-rings provide a new approach for miniaturizing devices. By optimizing the design of the resonator, stronger local enhancement effects can be achieved, thereby enhancing the interaction between light and electric field, and significantly improving modulation efficiency, thus facilitating compact optical modulation in small-sized devices. Resonant modulators can achieve high modulation efficiency in small sizes because the optical field circulates within the device and interacts multiple times with the electric field from external voltages.

### 5.1. Bragg Gratings

An optical Bragg grating is a transparent device characterized by a periodic modulation of the refractive index. This modulation results in a substantial reflectance, or reflectivity, within a specific wavelength range, known as the bandwidth, centered around a particular wavelength that satisfies the Bragg condition [53,54]:

$$\lambda_{B}=2\Lambda n_{eff}$$

Here, $\lambda_{B}$ represents the vacuum wavelength of light, $n_{eff}$ denotes the average refractive index of the medium, and Λ stands for the grating period. When this condition is satisfied, the wavenumber of the grating aligns with the discrepancy between the wavenumbers of the incident and reflected waves.

### 5.2. Microring Resonator

A typical optical microring resonator consists of a closed loop waveguide and a coupling mechanism to couple in/out the light. Resonance occurs when the waves traveling within the loop accumulate a phase shift equivalent to an integer multiple of 2π, resulting in constructive interference and resonance within the cavity. Assume that the reflections from the ring loop to the bus waveguide are negligible, the transmitted light field is expressed as follows:

$$\frac{E_{throught}}{E_{0}}=e^{j(\pi +\phi )}\frac{a-re^{-j\phi }}{1-rae^{j\phi }}$$

Here, ϕ=βL is the phase shift after one loop, *L* denotes the microring length, β is the propagation constant of the supported modes, and a represents the transmission amplitude and relates to the loss: $a^{2}=e^{-\alpha L}$. *r* is the self-coupling coefficient. The pass transmission of the bus waveguide is then written as:

$$T_{through}=\frac{a^{2}-2ra\cos\phi +r^{2}}{1-2ra\cos\phi +{\left(ra\right)}^{2}}$$

The ring is in resonance when the phase ϕ is an integer multiple of 2π. Then, the frequency of the light fits an integer number of times inside the effective length of microring:

$$\lambda_{res}=\frac{n_{eff}L}{m},m=1,2,3\ldots$$

The transmission is zero at critical coupling when the coupled light is totally attenuated inside the microring with r=a. Microring resonators serve as fundamental elements in high-index contrast photonic platforms, facilitating on-chip field enhancement, spectral filtering, and rapid modulation of optical signals. Over the last decade, microring resonators have been effectively showcased in various platforms, notably including LNOI.

**Figure 6:**
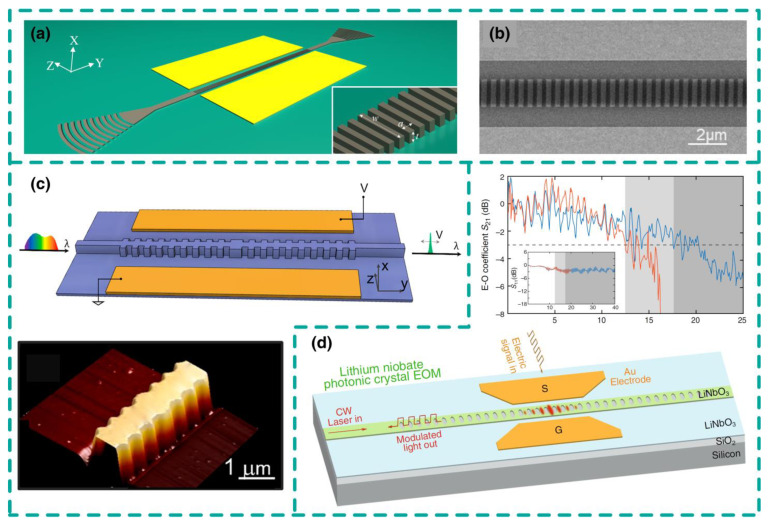
Resonant LNOI modulators. (**a**,**b**) Bragg gratings modulator based on the xcut LNOI platform. (**c**) MZI modulator based on slow-light Bragg gratings on two arms, showing large modulation efficiency V_π_·L = 0.67 V·cm and a compact footprint of 0.3 mm × 1.2 mm. (**d**) PhC modulator on LNOI platform, showing a modulation bandwidth of 17.5 GHz with a tiny volume of 0.58 μm^3^. (**a**,**b**) Adapted from [55], (**c**) adapted from [56], (**d**) adapted from [57].

Yaocheng Shi’s group utilized the principle of photonic bound states in the continuum in continuous media to fabricate photonic crystal nano-cavities on the LNOI platform (see Figure 6a,b). The device length is approximately 100 μm, with a quality factor exceeding 10,000. Due to the use of photoresist as the waveguide, there is no need to etch lithium niobate, significantly reducing processing difficulty [55]. Katia’s group proposed a slow-light structure of Bragg grating waveguide, as shown in Figure 6c, achieving a transmission bandwidth as narrow as 8.8 pm using phase-shifted Bragg grating filters [56]. Qiang Lin’s group at the University of Rochester designed and fabricated high-quality factor one-dimensional photonic crystal nano-resonators on LNOI (see Figure 6d). The device has a bandwidth of 17.5 GHz, with an electro-optic mode volume of only 0.58 μm^3^, achieving miniaturized modulators at the wavelength scale level on the LNOI platform for the first time [57]. Regarding micro-ring modulators, Marko Loncar’s team at Harvard University fabricated micro-ring electro-optic modulators on LNOI, achieving modulation bandwidths exceeding 10 GHz [58]. Liu Liu’s group at Zhejiang University introduced the MZI structure into the coupling region of micro-ring modulators, further improving their bandwidth while retaining the advantages of small half-wave voltage and device size [59]. Table 4 summarizes the performance of resonant LNOI modulators. Resonant LNOI modulators are typically limited by the trade-off between modulation efficiency and operating bandwidth, making it difficult to achieve high-speed, high-efficiency electro-optic modulation. They are usually only used for applications with high-quality factor requirements and relatively low bandwidth requirements, such as optical switch networks [60] and optical beam polarization [61].

## 6. Conclusions

In conclusion, we have demonstrated that thin-film LN modulators offer a promising solution for next-generation telecommunication systems and optical networks, largely due to their comprehensive performance in power consumption, bandwidth, and compact size, which are critical metrics for large scale photonic integrations. Despite not being the smallest in footprint or possessing the highest electro-optic efficiency compared to other promising material platforms like polymers, plasmonics, or barium titanate, LN offers well-balanced material properties and adequate integrability with decades of industry-proven operation. Next-generation photonic integration circuits require ultrahigh bandwidth, low driven voltage, and compact footprint, all of which thin-film LN is highly qualified. While intense research is ongoing to determine the most effective way to integrate thin-film LN with the rest of the photonics modality when considering techno-economic constraints, the outlook for fully realized thin-film LN circuits is unparalleled. The combination of low-loss passive optics, electro-optic, acousto-optic, and nonlinear optic functionalities on a monolithic material platform provides an incredibly powerful toolbox that we are only just beginning to explore.

## 7. Future Advancements

### 7.1. Fabrication Technologies

Future advancements in material preparation, such as the production of less expensive and higher quality wafers or the utilization of stoichiometric LN, hold the potential to significantly enhance device performance. Likewise, improvements in device fabrication processes, including etching, poling, doping, and annealing, will play a crucial role. Additionally, advancements in system integration, particularly in optical and radio frequency (RF) packaging, are essential for realizing immediate enhancements in device performance.

### 7.2. Loss Control

Further studies on photorefraction, charge accumulation, power handling, and loss mechanisms are urgently needed. These investigations are essential for a wide range of applications, including the achievement of extreme optical nonlinearities and the operation of electro-optic (EO) devices at high power, in harsh environments, and with long-term stability.

### 7.3. Linearity

The nonlinearity of the LN modulator arises when driven by large microwave sources, thus challenging the signal integrity in analog datalinks and multilevel photonic systems. Future linearity improvements include link loss control and complex linear modulation such as ring-assisted MZI [69] and cascaded MZI [70] in the LNOI platform.

## Figures and Tables

**Table 1:** Summary of photonic properties of representative materials for modulators.

Material	Optical Refractive Index	EO Coefficient (pm/V)	Second-Order Nonlinear Coefficient (pm/V)	RF Dielectric Constant
LiNbO_3_	2.21 (o) 2.14 (e)	r_13_ = 9.6 r_22_ = 6.8 r_33_ = 30.9 r_51_ = 32.6	d_31_ = −4.3 d_33_ = −27.0 d_22_ = 2.1	ε_11,22_ = 44 ε_33_ = 27.9
Si	3.48	0	0	11.7
SiO_2_	1.44	0	0	3.9
Si_3_N_4_	2	0	0	7.5
AlN	2.12 (o) 2.16 (e)	r_13_ = 0.67 r_33_ = −0.59	d_31_ = −1.6 d_33_ = −4.7	8.6
GaAs	3.38	r_41_ = 1.43	d_36_ = 170	12.9
LiTaO_3_	2.119 (o) 2.123 (e)	r_13_ = 8.4 r_22_ = −0.2 r_33_ = 30.5 r_51_ = 20	d_31_ = 0.85 d_33_ = 13.8	ε_11,22_ = 38.3 ε_33_ = 46.2

**Table 3:** Performance of monolithic LNOI modulators.

Modulator Type	V_π_·L (V∙cm)	3 dB Bandwidth (GHz)	Optical Propagation Loss (dB/cm)
Intensity Modulator [46]	2.7	175	<0.5
Intensity Modulator [49]	3.12	56	-
Intensity Modulator [1]	2.2	100	0.3
Intensity Modulator [50]	2.2	20	0.5
Intensity Modulator [51]	1.748	47	7
Intensity Modulator [52]	3.5	>45	0.5
IQ modulator [45]	2.47	48	0.15

**Table 4:** Performance of resonant LNOI modulators.

Modulator Type	Materials	Modulation Efficiency (pm/V)	Loaded Quality Factor
Microring Modulator [27]	LNOI	1.05	4 × 10^3^
Microring Modulator [62]	LN on SOI	1.7	1.7 × 10^4^
Microring Modulator [63]	LN on SOI	12.5	1.1 × 10^4^
Microring Modulator [64]	LN on SOI	3.3	1.2 × 10^5^
PhC Modulator [65]	Si on bulk LN	~2	1.2 × 10^5^
Racetrack Modulator [58]	LNOI	7	5 × 10^4^
Microring Modulator [66]	LNOI	4	2 × 10^6^
Microring Modulator [67]	SiN on LN	1.8	~9 × 10^4^
PhC Modulator [57]	LNOI	16	1.34 × 10^5^
Microring Modulator [68]	LNOI	2.15	2.8 × 10^3^

## Data Availability

The data presented in this study are available in the article.
